# A protein domain interaction interface database: InterPare

**DOI:** 10.1186/1471-2105-6-207

**Published:** 2005-08-25

**Authors:** Sungsam Gong, Changbum Park, Hansol Choi, Junsu Ko, Insoo Jang, Jungsul Lee, Dan M Bolser, Donghoon Oh, Deok-Soo Kim, Jong Bhak

**Affiliations:** 1 National Genome Information Center (NGIC), KRIBB, Daejeon, Korea; 2 Biomatics Lab, Dept. of BioSystems, KAIST, Daejeon, Korea; 3 Geometric Computing Lab, Division of Computer Science, KAIST, Daejeon, Korea; 4 Laboratory of Computational and Cellular Biology, Dept. of BioSystems, KAIST, Daejeon, Korea; 5 MRC-DUNN, Cambridge, UK; 6 Hanyang University, Seoul, Korea; 7 Object Interaction Technologies, Inc., Daejeon, Korea; 8 BiO center, Daejeon, Korea

## Abstract

**Background:**

Most proteins function by interacting with other molecules. Their interaction interfaces are highly conserved throughout evolution to avoid undesirable interactions that lead to fatal disorders in cells. Rational drug discovery includes computational methods to identify the interaction sites of lead compounds to the target molecules. Identifying and classifying protein interaction interfaces on a large scale can help researchers discover drug targets more efficiently.

**Description:**

We introduce a large-scale protein domain interaction interface database called InterPare . It contains both inter-chain (between chains) interfaces and intra-chain (within chain) interfaces. InterPare uses three methods to detect interfaces: 1) the geometric distance method for checking the distance between atoms that belong to different domains, 2) Accessible Surface Area (ASA), a method for detecting the buried region of a protein that is detached from a solvent when forming multimers or complexes, and 3) the Voronoi diagram, a computational geometry method that uses a mathematical definition of interface regions. InterPare includes visualization tools to display protein interior, surface, and interaction interfaces. It also provides statistics such as the amino acid propensities of queried protein according to its interior, surface, and interface region. The atom coordinates that belong to interface, surface, and interior regions can be downloaded from the website.

**Conclusion:**

InterPare is an open and public database server for protein interaction interface information. It contains the large-scale interface data for proteins whose 3D-structures are known. As of November 2004, there were 10,583 (Geometric distance), 10,431 (ASA), and 11,010 (Voronoi diagram) entries in the Protein Data Bank (PDB) containing interfaces, according to the above three methods. In the case of the geometric distance method, there are 31,620 inter-chain domain-domain interaction interfaces and 12,758 intra-chain domain-domain interfaces.

## Background

Proteins are the most important class of molecules in a cell. Most proteins function by interacting with other molecules, especially other proteins. The interactions among proteins are highly regulated and tightly conserved throughout evolution, [1,2] mainly because unnecessary or unsatisfactory interaction (misinteraction) triggered by random mutations can lead to molecular dysfunction. Therefore, interaction interface regions are under pressure from natural selection and are more conserved [3] compared to other exposed non-interface regions of proteins. Protein "structural interactomics" to map all the protein domain interactions is becoming increasingly important as more complete genome sequences are made available [4-7]. Now scientists can map the whole human interactome bioinformatically [8], using ever-increasing experimental data coming from methods such as yeast two-hybrid analysis. Consequently, a higher resolution molecular interaction analysis is also becoming more important.

Since the 1970s, there has been much effort to determine the principles of protein-protein recognition. Pioneers in the field of protein-protein interaction, such as Chothia and Janin [9], have studied the physical and chemical properties of protein interaction sites that contribute to the recognition processes. Colman *et al*. [10,11] focused on electrostatic and shape complementarity of interaction interfaces using EC (Electrostatic Complementarity) and shape correlation index, respectively. Argos [12] studied interfaces between protein subunits or protein domains. He not only investigated the physicochemical properties of protein interfaces, but also tried to understand the geometric features of protein interfaces using a spline function [13,14]. Jones and Thornton [15] introduced a surface patch method to find out the parameters that contribute to the process of protein-protein interaction. Chakrabarti and Janin [16,17] investigated the structure of interface region by dissecting it into core and rim based on different solvent accessibility. They also addressed the chemical properties of each region.

Recently, there has been a new trend in the study of protein interfaces. Several groups have introduced computational geometric and topology methods for the study of protein interfaces. Most importantly, the Voronoi diagram [18,19,23] has been used to study interfaces of protein complexes. As early as 1974, Richards [20,21] first introduced the Voronoi diagram as an application for protein structure study, although not specifically as an interface analysis tool.

Despite all the efforts to unveil the underlying principles of protein-protein interaction for over 30 years, there has not been much progress at the fundamental level since the research by Chothia and Janin [9]. The interface data derived from different approaches are not well maintained or widely shared amongst scientists. Fortunately, with the help of faster X-ray crystallography and NMR in structural biology, there has been an increase in the number of known three-dimensional protein structures. This 3D structure information is a good source of data for the study of protein interfaces.

Here, we introduce a large-scale protein interaction interface database called InterPare ( or ). InterPare presents interfaces between protein domains identified by three methods. First, the interface is detected by calculating the geometric distance between subunits of multidomain proteins or protein complexes in the PDB [22,27]. In the second approach, buried protein regions are identified by calculating the accessible surface area (ASA) when they form a complex or an aggregate with other subunits or domains. These buried regions can be accessible to water when they are in a free subunit or one domain state. Finally, interfaces are defined by a geometric and topological approach using the Voronoi diagram [18,19,23]. InterPare presents protein interfaces defined by the Voronoi diagram. The interface structure of queried proteins, in the context of the whole protein configuration, can be viewed with three different molecular viewers on the results page. They are the Chime [24], Jmol [25], and InterFacer [26]. InterPare also provides the atomic coordinate files for protein surface, interior, and interface for further analysis.

## Construction and content

### Data sets

**Figure 1:**
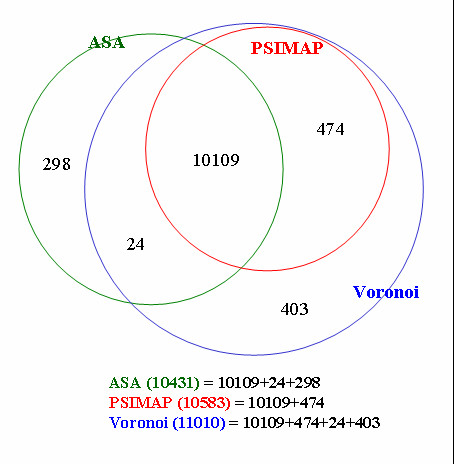
PDB data coverage and overlap of three interface identification methods. Numbers in a circle represent the number of PDB entries whose interfaces have been identified according to ASA (green), PSIMAP (red), and Voronoi (blue) methods. In the case of multidomain proteins, 24, 474, and 403 PDB entries are exclusively identified by ASA, PSIMAP, and Voronoi method, respectively. The main reason for the exclusive detection results from the different method of interface identification. Interfaces from 298 PDB entries, uniquely identified by the ASA method, are not domain-domain interfaces. They are interfaces between a domain and DNA (RNA), or between a domain and non-domain region from a different chain. The numbers are based on SCOP 1.65. All the PDB entries that belong to each category are downloadable on the InterPare website.

Proteins in the PDB [22,27] were used to investigate interacting interfaces of protein domains. For a domain definition, we used the Structural Classification of Proteins (SCOP) [28,29]. As of this writing, InterPare uses SCOP 1.65 which is based on around 20,600 PDB entries. The ASTRAL compendium [30,31] provides 3D coordinate files of domains in SCOP. InterPare contains 10,583, 10,431, and 11,010 PDB entries that have been identified as containing interacting interfaces according to geometric distance, ASA, and the Voronoi diagram methods (see interface identification methods below) respectively. Figure 1 shows the extent of PDB data sets covered by each method and their overlap according to the three methods. Interfaces from 10,109 PDB entries can be commonly identified by these three methods. All the interfaces derived by the geometric distance method (green) can also be detected by the Voronoi method (blue) because the latter covers all the multidomain proteins in SCOP (11,010 PDB entries based on SCOP 1.65) by using a mathematical definition of interfaces. The three interface identification methods are explained in the following section.

### Interface identification methods

We identified interaction interfaces of protein domains by:

1) Calculating the geometric distance between atoms in different domains (PSIMAP method).

2) Detecting the differences of Accessible Surface Area (ASA) from all the residues in two states: the detached individual subunit state and the multimeric state.

3) Calculating Voronoi diagrams.

1. The geometric distance method checks the distance between atoms in two interacting domains.

Two domains are assumed to interact with each other if there are at least 5 residue pairs whose atomic distance falls within 5 Angstrom distance (5-5 rule), according to the PSIMAP algorithm [32-34]. In this method, domain-domain interaction interfaces are defined as a set of atoms satisfying the threshold of the 5-5 rule by using FAC PSIMAP method [35]. We define an amino acid residue as an interface residue if its atoms are within the threshold 5 Angstrom is a threshold based on Van der Waals radii of interacting atoms and a solvent such as water. The distance threshold (5 Å is a default) can be varied by users on the website. As the threshold gets higher the number of interface residue gets smaller. We used SCOP 1.65 as a domain definition. It contains 54,745 domains from 20,619 PDB Entries (August 2003). InterPare, at the time of this writing, contains 26,999 PDB entries (September 2004). At present, there is a faster algorithm available that uses the convex hull concept [36]. However, the present C program was efficient enough in that it took only 15 hours to complete the calculation for all the entries in the PDB. It is based on a distributed linux cluster system with 22 computing nodes each of which has Intel Xeon 3.0 GHz CPU and 2 GB memory. Current PSIMAP program can be freely downloadable from the PSIMAP website [37].

2. The Accessible Surface Area (ASA) method detects protein regions that are buried and hence excluded from a solvent when forming a multimer or a complex.

If two or more subunits form a protein complex or aggregate, they have to lose a portion of area that was accessible by a solvent (typically water). With the ASA method, we define interface residues as residues that have lost more than 1 Å^2 ^solvent accessible surface area (ASA) upon aggregation or complexation [15,38,39]. It can be formulated as follows.



**Figure 2:**
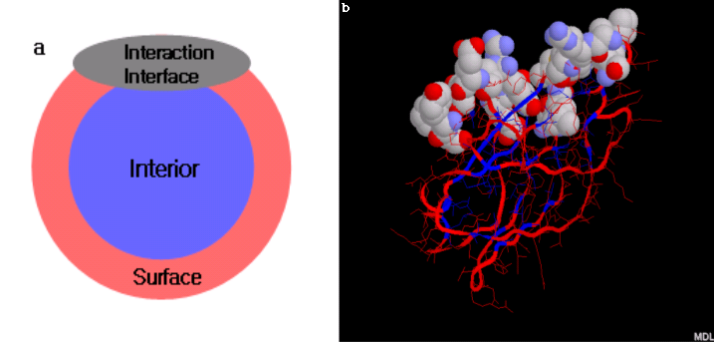
Protein structure with respect to their geometrical region. **(a) **Schematic diagram representing the interior, interface, and surface of longitudinal section of a protein domain. **(b) **An example of a 3D structure (SCOP id: d1a25a_) which corresponds to a schematic diagram (a). It shows the three areas of a domain (red: protein surface, blue: protein interior, filled-in space model: interaction interface). Interface regions are represented as a space-fill model to distinguish them from other regions.

For all residues () in a SCOP domain and their corresponding residues () in a PDB entry,  and  can be either an interface residue (*Interface*(, ) = 1) or a non-interface residue (*Interface*(, ) = 0) based on the difference of ASA in that residue. The threshold (1 Å^2 ^in our case) can be selected by the user on the InterPare website (from 1 Å^2 ^to 5 Å^2^). As the threshold gets higher, the number of interface residues gets smaller. An interface region, in a domain, that consists of at least 10 interface residues is acceptable, and those having less than 10 residues are considered as artifacts. InterPare only serves domain interaction interfaces having at least 10 interface residues. We calculated the ASA of protein molecules using a program called NACCESS [40,41], an implementation of the algorithm developed by Lee and Richards [42]. It calculates the absolute ASA and the relevant ASA in terms of total residues, side chains, polar atoms, and non-polar atoms. Relative accessibilities, for each residue in a domain or a protein, can be expressed as the ratio of the surface area of a residue in an intact state to that of a residue in an Ala-X-Ala tri-peptide state [43]. Surface residues are defined as those that have a relative ASA of more than 5% [44]. Interior residues are defined as those that have a relative ASA of less than 5%. This threshold can also be chosen on the InterPare website. The default van der Waals radii of atoms were taken from Chothia [43]. We used water of 1.40 van der Waals radii as a solvent. In Figure 2, a protein domain is shown which is divided into three regions (interface, interior, surface) according to the ASA method.

3. The Voronoi diagram, also known as Dirichlet Tessellation, has been widely used in the fields of science and engineering. The Voronoi diagram was first introduced as an application for the study of protein structures by Richards [20,21]. There is a report on defining molecular interfaces by Power Diagram; Voronoi Diagram on a weighted point set [45]. We used the same protocol suggested by Varshney *et al*. [45], but applied our own polygon filtering method and calculated interfaces only between domains instead of calculating them on protein complexes.

**Figure 3:**
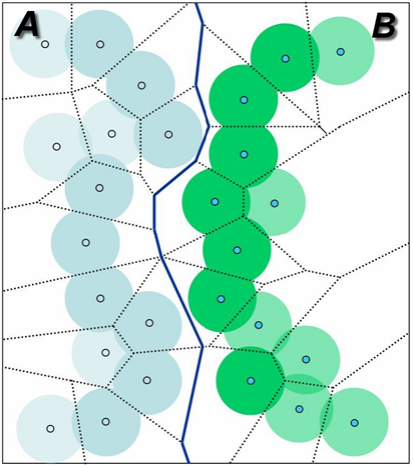
Power diagram of two different domains in 2D representation. Light blue circles (atoms) are contained in domain A, and green atoms are in domain B. Dotted lines denote Voronoi edges between two neighboring atoms, and solid lines represent the Voronoi geometrical interface between two domains. Any polygon which is adjacent to at least one Voronoi geometrical interface is called an interface-cell. If a cell is an interface-cell, then we call the atom in the cell an interface-atom. Interface-atoms are slightly darker than non-interface atoms. The InterPare database stores all interface-atom information.

First of all, a three dimensional power-diagram *P *of the atoms was constructed. Each face of the power-diagram *P *is defined by two adjacent atoms (Figure 3). Power-diagrams generate polygons which are bounded by edges. An edge, represented as a blue solid line in Figure 3, is defined by two atoms each of which belongs to different domains. The construction of such a power diagram, in an average case, will have a time complexity of *O*(*n*) (*n *is number of atoms in the protein) [46,47] where the number of neighbors for any given atom is bounded by a constant.

To have polygons only close to the interaction region, marginal polygons need to be filtered out because those are irrelevant to the interacting interfaces. We removed all the marginal polygons by using our two-stage polygon filtering method. At first stage, we removed polygons which do not contain edges defined by interface atoms. Interface atoms are those in the interface residue defined by ASA method (see above). The default van der Waals radii of atoms were taken from Chothia [43]. Polygons are further filtered out if they have one or more vertices which are beyond 5 Angstrom distance from the interface atoms. For each face in *P *(Figure 3), if two atoms defining a face belong to different domains from each other, we call such a face an interface-face. Let us define interface-cells as cells in the power-diagram *P *that have at least one interface-face. Let us define interface-atoms to be those atoms whose cells are interface-cells. In the InterPare database, all the interface-atoms between two domain pairs are stored in a PDB-style file format.

## Utility

**Figure 4:**
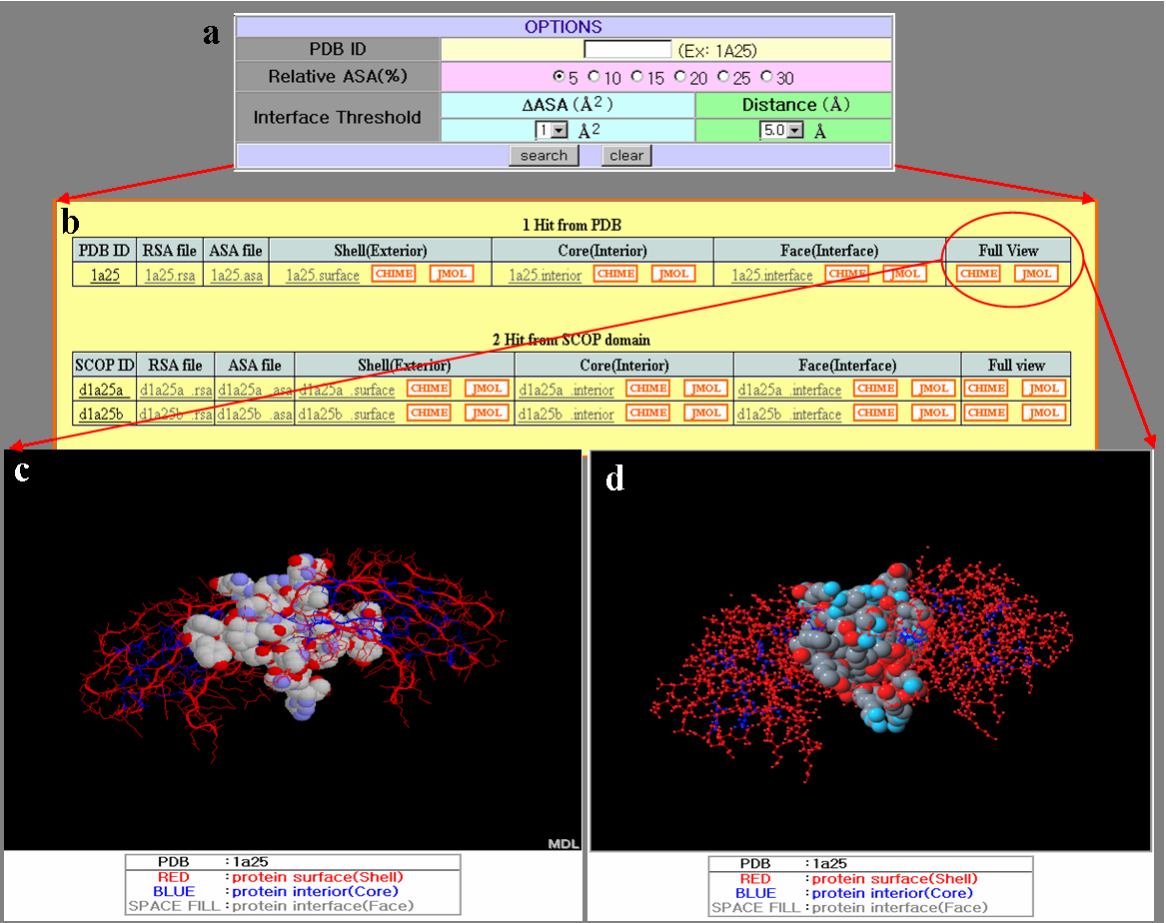
Screen shots from the results page of the InterPare web site. **(a) **The query box with several options. 'Relative ASA' is a criterion for discerning the interior region of a protein from its surface region. There are two independent criteria for the interface definition: 'ΔASA' for the ASA method, and 'Distance' for the PSIMAP algorithm. **(b) **Results of querying the database with '1a25' as a PDB ID. 1a25 is a C2 domain from the protein kinase C beta. It consists of two identical domains (d1a25a_ and d1a25b_). Results by the ASA method are shown here. The atomic coordinates for the protein surface (Shell), interior (Core), and interface (Face) regions are downloadable on this page. **(c)**, **(d) **The 3D structure of protein '1a25' by Chime and Jmol, respectively. Interior regions are blue and surface regions are red. Interface regions are displayed in space-fill model and other non-interface regions are displayed in wireframe with a backbone tracing mode.

InterPare contains protein surface, interior, and interface information from PDB entries. There are three query interfaces to access the information in InterPare. Queries can be 1) keywords, 2) PDB or SCOP IDs, or 3) protein sequences in FASTA format. In the case of a protein sequence, InterPare provides a structural domain assignment module using PDB-ISL [48] and PSI-BLAST [49,50] to assign homologous domains in SCOP to the queried sequence. All the queries are finally assigned to (a) PDB ID(s). Figure 4a shows the search interface in the case of a PDB ID as a query. Relative ASA (see interface identification methods above), in Figure 4a, is a criterion for the protein interior and surface boundaries. There are two options for the interface definition threshold: one for the geometric distance method, and another for the ASA method (See the interface identification method above for the threshold criteria). Figure 4b shows the results of PDB ID '1a25' as a query by the ASA method. It contains protein surface, interior, and interface information. We implemented Chime [24] and Jmol [25] scripts to let users view protein 3D structures in a pop-up window when the links are clicked, as in Figure 4c and 4d. Protein surfaces and interiors are in red and blue, respectively, and the interface is viewed in space-fill mode to distinguish it from other parts of the protein molecule. To view protein structures, the Chime plug-in and a Java runtime environment with Java 3D 1.3.1+ are required. The InterFacer homepage  provides files that are required to view molecules with InterFacer. Atom coordinate files of three different regions are available to download. In addition, 1) the size of the interface and surface area, and 2) amino acid compositions on the surface, interior, and interface regions are provided on the results page.

## Discussion

The protein interfaceome can be defined as the whole set of protein interaction interfaces found in cells. There can be many methods to define such an interface data set. We use the concept of the hierarchical classification of protein domains from SCOP. We extend the SCOP classification to molecular interfaces. The advantage of this approach is that each interface can be classified in the context of domain evolution. SCOP Superfamily is the level of classification where protein structures are clearly known to be related within the classification group. The protein Family level in SCOP is a more functionally relevant class, where each member of the Family is related and functionally similar. Below Family, there are individual domains. We applied three algorithms to find interfaces associated with SCOP. Any protein domain classification system, such as FSSP [51] and CATH [52], can also be used. The main contribution to structural bioinformatics is that interfaces can be searched and compared (hence InterPare) by computer.

We expect that hierarchically similar clusters in the interfaceome will have highly conserved interfaces to maintain their interaction partners. This can provide a new level of functional prediction capability for the designing of novel molecules that can interface with proteins and hence control protein activities.

## Conclusion

InterPare is an open and public database server for protein interaction interface information. It contains large-scale interface data for proteins whose 3D-structures are known. We identified 31,620 inter-chain interfaces and 12,758 intra-chain interfaces. At this moment, there are 10,583, 10,431, and 11,010 PDB entries whose domain interaction interfaces have been identified according to geometric distance, ASA, and Voronoi diagram methods, respectively. These interfaces are based on protein domains which are from the SCOP database. By using SCOP, InterPare is tightly associated with the domain classification hierarchy, making the search and lookup convenient.

## Availability and requirements

InterPare is available through . InterPare is jointly maintained by the National Genome Information Center (NGIC) of Korea, Object Interaction Technologies, Inc., Daejeon, Korea, and the BiO Center . It is free to any user.

## List of abbreviations used

PSIMAP: Protein Structural Interactome map

PDB: Protein Data Bank

SCOP: Structural Classification Of Protein structure

FSSP: Fold classification based on Structure-Structure alignment of Proteins

## Authors' contributions

SSG worked on the ASA part, drafted the manuscript, and managed this project. CBP implemented a program regarding the Voronoi diagram. JSK developed the InterPare webpage. ISJ and DMB identified protein interfaces using the PSIMAP algorithm. HSC implemented molecular viewer named InterFacer. JSL made C version of PSIMAP program. DSK supervised the development of Voronoi diagram method. DHO and JB supervised this project and revised the manuscript. All authors have read and accepted the final manuscript.

## References

[B1] Bolser DM, Park J (2003). Biological network evolution hypothesis applied to protein structural interactome. Genomics and Informatics.

[B2] Park J, Bolser D (2001). Conservation of Protein Interaction Network in Evolution. Genome Informatics.

[B3] Caffrey DR, Somaroo S, Hughes JD, Mintseris J, Huang ES (2004). Are protein-protein interfaces more conserved in sequence than the rest of the protein surface?. Protein Science.

[B4] Kim WK, Bolser DM, Park JH (2004). Large scale co-evolution analysis of Protein Structural Interlogues using the global Protein Structural Interactome Map (PSIMAP). Bioinformatics.

[B5] Bolser DM, Dafas P, Harrington R, Park J, Schroeder M (2003). Visualisation and graph-theoretic analysis of a large-scale protein structural interactome. BMC Bioinformatics.

[B6] Park D, Lee S, Bolser D, Schroeder M, Lappe M, Oh D, Bhak J (2005). Comparative interactomics analysis of protein family interaction networks using PSIMAP (protein structural interactome map). Bioinformatics.

[B7] Moon HS, Bhak J, Lee KH, Lee D (2005). Architecture of basic building blocks in protein and domain structural interaction networks. Bioinformatics.

[B8] Kim HG, Park J, Han KS (2003). Predicting Protein Interactions in Human by Homologous Interactions in Yeast. Lecture Notes in Computer Science.

[B9] Chothia C, Janin J (1975). Principles of protein-protein recognition. Nature.

[B10] McCoy AJ, Epa VC, Colman PM (1997). Electrostatic Complementary at Protein/Protein Interfaces. J Mol Biol.

[B11] Lawrence MC, Colman PM (1993). Shape complementarity at protein/protein interfaces. J Mol Biol.

[B12] Argos P (1998). An investigation of protein subunit and domain interfaces. Protein Eng.

[B13] Harder RL, Desmarais RN (1972). Interpolation Using Surface Splines. J Aircraft.

[B14] Meinguet J (1979). Multivariate Interpolation at Arbitrary Points Made Simple. J Appl Math Phys.

[B15] Jones S, Thornton JM (1997). Analysis of Protein-protein interaction sites using surface patches. J Mol Biol.

[B16] Chakrabarti P, Janin J (2002). Dissecting Protein-Protein Recognition Sites. Proteins.

[B17] Bahadur RP, Chakrabarti P, Rodier F, Janin J (2003). Dissecting Subunit Interfaces in Homodimeric Proteins. Proteins.

[B18] Ban YEA, Edelsbrunner H, Rudolph J (2004). Interface surfaces for protein-protein complexes. Proceedings of the Research in Computational Molecular Biology, San Diego.

[B19] Poupon A (2004). Voronoi and Voronoi-related tessellations in studies of protein structure and interaction. Curr Opin Struct Biol.

[B20] Richards FM (1974). The interpretation of protein structures: total volume, group volume distributions and packing density. J Mol Biol.

[B21] Richards FM (1977). Area, volumes, packing and protein structures. Ann Rev Biophys Bioeng.

[B22] Protein Data Bank. http://www.rcsb.org/pdb.

[B23] Kim DS, Cho YS, Kim DG, Kim SS, Bhak J, Lee SH (2005). Euclidean Voronoi Diagrams of 3D Spheres and Applications to Protein Structure Analysis. Japan Journal of Industrial and Applied Mathematics.

[B24] Chime. http://www.mdl.com/products/framework/chime.

[B25] Jmol. http://jmol.sourceforge.net.

[B26] InterFacer. http://www.interfacer.org.

[B27] Berman HM, Westbrook J, Feng Z, Gilliland G, Bhat TN, Weissig H, Shindyalov IN, Bourne PE (2000). The Protein Data Bank. Nucleic Acid Res.

[B28] Structural Classification Of Proteins. http://scop.mrc-lmb.cam.ac.uk/scop.

[B29] Murzin AG, Brenner SE, Hubbard T, Chothia C (1995). SCOP: a structural classification of proteins database for the investigation of sequences and structures. J Mol Biol.

[B30] ASTRAL. http://astral.berkeley.edu.

[B31] Brenner SE, Koehl P, Levitt M (2000). The ASTRAL compendium for sequence and structure analysis. Nucleic Acids Res.

[B32] Park J, Lappe M, Teichmann S (2001). Mapping Protein Family Interactions: Intramolecular and Intermolecular Protein Family Interaction Repertoires in the PDB and Yeast. J Mol Biol.

[B33] Han KS, Park BK, Kim HG, Hong JS, Park J (2004). HPID: The Human Protein Interaction Database. Bioinformatics.

[B34] Lappe M, Park J, Niggemann O, Holm L (2001). Generating protein interaction maps from incomplete data: application to Fold assignment. Bioinformatics.

[B35] Gong SS, Yoon GS, Jang IS, Bolser DM, Dafas P, Schroeder M, Choi HS, Cho YB, Han KS, Lee SH, Choi HH, Lappe M, Holm L, Kim SS, Oh DH, Bhak JH (2005). PSIbase: a database of Protein Structural Interactome map (PSIMAP). Bioinformatics.

[B36] Dafas P, Bolser DM, Gomoluch J, Park J, Schroeder M (2004). Using convex hulls to extract interaction interfaces from known structures. Bioinformatics.

[B37] PSIMAP. http://psimap.org.

[B38] Jones S, Thornton JM (1996). Principles of protein-protein interactions. Proc Natl Acad Sci.

[B39] Jones S, Marin A, Thornton JM (2000). Protein domain interfaces: characterization and comparison with oligomeric protein interfaces. Protein Engineering.

[B40] NACCESS. http://wolf.bms.umist.ac.uk/naccess.

[B41] Hubbard SJ, Thornton JM (1993). NACCESS.

[B42] Lee B, Richards FM (1971). The Interpretation of Protein Structures: Estimation of Static Accessibility. J Mol Biol.

[B43] Chothia C (1976). The nature of the accessible and buried surfaces in proteins. J Mol Biol.

[B44] Miller S, Janin J, Lesk AM, Chothia C (1987). Interior and surface of monomeric proteins. J Mol Biol.

[B45] Varshney A, Brooks F, Richardson D (1995). Defining, Computing, and Visualizing Molecular Interfaces. IEEE Visualization.

[B46] Halperin D, Overmars MH (1994). Spheres, Molecules, and Hidden Surface Removal. The Proceedings of the 10th Annual ACM Symposium of Computational Geometry.

[B47] Dwyer RA (1989). Higher-Dimensional Voronoi Diagrams in Linear Expected Time. The Proceedings of the 5th Annual ACM Symposium on Computational Geometry.

[B48] Teichmann SA, Chothia C, Church GM, Park JH (2000). Fast assignment of protein structures to sequences using the intermediate sequence library PDB-ISL. Bioinformatics.

[B49] BLAST. http://www.ncbi.nlm.nih.gov/BLAST/.

[B50] Altschul SF, Madden TL, Schaffer AA, Zhang J, Zhang Z, Miller W, Lipman DJ (1997). Gapped BLAST and PSI-BLAST: a new generation of protein database search programs. Nucleic Acids Res.

[B51] Holm L, Sander C (1996). Mapping the protein universe. Science.

[B52] Orengo CA, Michie AD, Jones S, Jones DT, Swindells MB, Thornton JM (1997). CATH – A Hierarchic Classification of Protein Domain Structures. Structure.

